# Comparative fMRI and MEG localization of cortical sensorimotor function: Bimodal mapping supports motor area reorganization in glioma patients

**DOI:** 10.1371/journal.pone.0213371

**Published:** 2019-03-07

**Authors:** Max Zimmermann, Karl Rössler, Martin Kaltenhäuser, Peter Grummich, Nadja Brandner, Michael Buchfelder, Arnd Dörfler, Konrad Kölble, Andreas Stadlbauer

**Affiliations:** 1 Department of Neurosurgery, University of Erlangen-Nürnberg, Erlangen, Germany; 2 Department of Neuroradiology, University of Erlangen-Nürnberg, Erlangen, Germany; 3 Department of Neuropathology, University of Erlangen-Nürnberg, Erlangen, Germany; 4 Institute of Medical Radiology, University Clinic of St. Pölten, St. Pölten, Austria; Boston Children’s Hospital / Harvard Medical School, UNITED STATES

## Abstract

**Introduction:**

Preoperative functional mapping in the vicinity of brain lesion is of high importance for avoiding complications in surgical management. However, space-occupying lesions may lead to functional reorganization or decreased BOLD activity.

**Methods:**

Therefore in 13 patients with cerebral gliomas or brain arterio-venous malformations/ hemangioma fMRI- and MEG-based cortical localizations of motor and somatosensory cortical activation pattern were compared in order to investigate their congruency.

**Results:**

Localization of cortical sensorimotor areas with fMRI and MEG showed good congruency with a mean spatial distance of around 10 mm, with differences depending on the localization method. The smallest mean differences for the centroids were found for MEF with MNE 8 mm and SEF with sLORETA 8 mm. Primary motor area (M1) reorganization was found in 5 of 12 patients in fMRI and confirmed with MEG data. In these 5 patients with M1-reorganization the distance between the border of the fMRI-based cortical M1-localization and the tumor border on T1w MR images varied between 0–4 mm, which was significant (*P* = 0.025) different to the distance in glioma patients without M1-reorganization (5–26 mm).

**Conclusion:**

Our multimodal preoperative mapping approach combining fMRI and MEG reveals a high degree of spatial congruence and provided high evidence for the presence of motor cortex reorganization.

## 1. Introduction

Neurosurgical procedures of lesions adjacent to eloquent brain areas are associated with a risk of postoperative dysfunction and always represent a challenge in neurosurgical decision making. Given the direct correlation between extent of tumor resection and clinical outcome, an exact mapping of sensorimotor (SM) areas in patients with perirolandic lesions can avoid irreversible impairment of SM function and facilitate a more radical resection, thus increasing both postoperative quality of life and survival time [1]. Therefore, preoperative characterization of the functional anatomy in the vicinity of the lesion is of high importance for effective surgical management. However, it is often difficult to depict the relation of the lesion to functional important structures [2], especially when edema or mass lesions are present that distort the local anatomy of the brain. Furthermore, space-occupying lesions may lead to functional reorganization and alter the topographic organization of the cortex [3,4]. Previous studies provided examples for lesion-induced brain plasticity leading to considerable regional shifts of functional brain areas [5–11]. However, lesion-induced transhemispheric cortical reorganization to homologous brain regions (homotopic reorganization) in adult patients is considered controversial in the scientific community [12].

Functional magnetic resonance imaging (fMRI) [13] is the method of choice for non-invasive presurgical localization of eloquent cortex areas in the vicinity of brain lesions. fMRI relies on the blood oxygen level dependent (BOLD) effect [14]. It is physiologically based on the regional vasoactive responses induced by neuronal activity, which increase regional cerebral blood flow (CBF) and blood oxygen concentration. The BOLD effect, however, can be significantly compromised by the presence of glial tumors, both at the edge of the tumor and in vascular territories somewhat remote from the tumor, due to loss of regional cerebral vasoactivity [15–18].

Magnetoencephalography (MEG), however, more directly detects neuronal brain activity by measuring magnetic field distribution of the whole brain. While both methods have a spatial resolution in the mm range, MEG provides a time resolution in milliseconds, in contrast to fMRI with a time resolution of 2–3 seconds. Due to its greater availability and robustness fMRI is more frequently used.

The clinical use of MEG is based on former studies having compared fMRI and MEG localizations of SM activation in tumor patients [19–22] or use of MEG for localization of eloquent brain areas in surgical planning [23–25]. Recently the combined use of fMRI and MEG prior to radical brain tumor resection at the precentral gyrus was shown to be effective in the preservation of motor function (Izutsu et al. 2017). Similarly integrating MEG and fMRI localizations into a CyberKnife treatment protocol can prevent eloquent brain areas from radiogenic damage [26].

This study was conducted to examine functional reorganization in patients with brain lesions using a multimodal approach. Furthermore we aimed to compare the precision of fMRI and MEG as well as of three commonly used MEG data postprocessing software tools in the presurgical evaluation of SM function.

## 2. Material and methods

### 2.1. Subjects

The study protocol was approved by the local ethics committee of the University of Erlangen-Nürnberg for MEG (Nr. 226_12B), MRT (Nr. 3578 and 177_14B) and was in line with the Helsinki Declaration of Human Rights. Thirteen patients (9 male, 4 female; mean age ± standard deviation, 49.5 ± 13.2 years; age range, 32–76 years) with brain lesions (close to the SM region) were consecutive admissions to the Department of Neurosurgery of the Friedrich Alexander University’s (FAU) medical center. Eleven patients had neuropathologically proven gliomas (seven glioblastoma WHO°IV, three anaplastic glioma WHO°III, one low-grade astrocytoma WHO°II) and two had arteriovenous malformations/hemangiomas (AVM/H) (Table 1). Seven patients demonstrated preoperative SM deficits. The patients preoperative functional status were in detail as follows: ID 1 had no SM deficits, ID 2 had no SM deficits despite tumoral compression of the pyramidal tract (PT) and the motor cortex (MC), ID 3 had minor motor deficits of the right hand due to tumoral infiltration and compression of the PT, ID 4 had no SM deficits despite tumoral compression of the PT and the MC, ID 5 had coordination problems with considerably tumoral compression of the PT, ID 6 had epileptic seizures due to tumoral infiltration and compression of the PT and the MC, ID 7 and ID8 had no SM deficits, ID 9 had epileptic seizures with tumoral infiltration of the PT, ID 10 had a discrete paresis of the left hand with tumoral infiltration of the premotoric area, ID 11 had minor motor deficits with compression of the PT and the MC, ID 12 had a discrete left hemiparesis with tumoral infiltration and compression of the PT and the MC, ID 13 had no SM deficits in spite of tumoral infiltration of the PT and the MC. Between 7 and 14 days postoperatively ID 8 showed a right hemiparesis due to a lesioned PT, ID9 had a discrete right hemiparesis and ID 10 has a discrete paresis of the left hand due to persistent tumoral infiltration of the premotoric area, the remaining ten of the thirteen patients were free of SM deficits.

**Table 1:** Clinical details of the patient cohort.

Patient			Lesion				Funct. Img.		SM Deficits		M1 dist. to		Reorg
ID	Sex	Age	Histopathology	Location	Vol.	IDH	fMRI	MEG	preop	postop	CE	native	M1
1	M	55	GB WHO°IV	r parietal	130,0	wt	no ^†^	yes	no	no	n/a	n/a	no
2	M	76	GB WHO°IV	r frontal	77,9	wt	yes	yes	no	no	20	2	yes
3	M	54	GB WHO°IV	r trigonal	58,1	wt	yes	yes	yes	no	24	9	no
4	M	41	Astro WHO°III	l parietal	71,7	mut	yes	yes	no	no	13	4	yes
5	M	59	GB WHO°IV	r temporal	231,9	wt	yes	yes	yes	no	31	13	no
6	M	43	OD WHO°III	r central	40,2	mut	yes	yes	yes	no	15	0	yes
7	F	36	AVM/H	r temporal	0,3	n/a	yes	yes	no	no	n/a	2	no
8	M	44	AVM/H ^¶^	l postcentral	7,9	n/a	yes	yes	no	yes	n/a	0	no
9	F	41	OD WHO°III	l frontal	67,0	mut	yes	yes	yes	yes	37	5	no
10	M	71	GB WHO°IV	r frontal	21,4	wt	yes	(yes) ^§^	yes	yes	36	26	no
11	F	32	Astro WHO°II	r parietal	29,3	mut	yes	(yes) ^§^	yes	no	n/a	11	no
12	F	51	GB WHO°IV	r frontal	15,7	wt	yes	yes	yes	no	31	2	yes
13	M	40	GB WHO°IV	l frontal	11,8	wt	yes	no^‡^	no	no	7	2	yes

^†^ Patient aborted the fMRI measurement;

^‡^ Patient refused the MEG measurement;

^§^ MEG data quality was insufficient due to magnetic artefacts from dental implants;

^¶^ Spetzler-Martin AVM grading system;

Abbreviations: M, male; F, female; Vol., native T1w MRI Volume in cm^3^; GB, glioblastoma; Astro, astrocytoma; OD, oligodendroglioma; AVM/H, arteriovenous malformation/hemangioma; r, right; l, left; IDH, isocitrate-dehydrogenase; wt, wildtype; mut, mutated; SM deficits, sensorimotor deficits; preop, preoperative; postop, postoperative; M1 dist. to, distance in mm between M1 area in fMRI and the tumor border on CE or native T1w MRI; CE, contract enhanced T1w MRI; native, T1w MRI without CE; n/a, not applicable; Reorg., reorganization of M1 detected.

The differences in patients’ cortical SM function localizations between the two modalities were compared to those obtained from three healthy volunteers (2 male, 1 female; mean age 39.0 ± 13.1 years; age range, 30–54 years).

### 2.2. MRI data acquisition

Anatomical and functional MRI data acquisitions were performed on a 1.5 Tesla clinical whole-body MR scanner (Magnetom Sonata, Siemens, Erlangen, Germany). The fMRI protocol included a 3D T1-weighted magnetization prepared rapid acquisition gradient echo (MPRAGE) sequence with the following parameters: field of view (FOV), 256 x 256 mm; acquisition matrix, 256 x 256; slice thickness, 1 mm; number of slices, 160; echo time (TE), 4.38 ms; repetition time (TR) 2090 ms; flip angel 15°. This data were used to generate individual anatomical reference data for fMRI and MEG postprocessing and localization.

For the fMRI experiments we used a conventional 2D echo planar imaging (EPI) sequence with the following parameters: FOV 192 x 192 mm, acquisition matrix 64 x 64, slice thickness 3 mm, number of slices 16, TE 60 ms, TR 2470 ms, flip angle 90°. A block paradigm with 180 measurements in 6 blocks (3 resting state and 3 active intervals) with 30 volumes each was used. During fMRI examination the patients were immobilized in a vacuum pad to avoid head movements. Prior to the motor measurements the patients were instructed to repeatedly flex and extend all digits (Exercise 1) or foot and toes (Exercise 2) of a designated side. Healthy volunteers and patients were asked to refrain from any other motor actions. Start and stop commands for the movements were given acoustically and the patients’ movements were monitored from the control room. Any deviation from protocol were noted and considered in the evaluation. In somatosensory measurements, the patients were instructed to avoid any motor activity and the stimulation of the index finger was started and stopped automatically according to the intervals.

Clinical MRI examinations were performed on a 3 Tesla clinical whole-body MR scanner (Tim Trio; Siemens, Erlangen, Germany) using a T1w sequence (fast low-angle shot, FLASH; FOV 230 x 230 mm, acquisition matrix 256 x 256, slice thickness 4 mm, TE 2.24 ms, TR 300 ms, flip angle 70°) for the determination of the contrast agent enhancement region. gadoterate meglumine (Dotarem, Guerbet) was used for contrast.

### 2.3. fMRI data evaluation

For motion correction we applied an image-based prospective acquisition correction interpolated in the k-space [27]. The linear correlation maps were computed and analyzed with respect to their signal intensities using a square wave reference function for each pixel (BrainVoyager, Brain Innovation, Maastricht, Netherlands).

Pixels exceeding a significance threshold (correlations above a threshold of 0.3 with p < 0.000045) [24] were displayed and clusters of at least 4 contiguous voxels were assembled in order to eliminate isolated voxels. Subsequently the functional data sets were fused to the anatomical data sets (T1w MRI data set with 1 mm^3^ isotropic voxels) and clustering was increased to display the fMRI results.

The threshold results from the following consideration: Using a matrix (64 * 64 * 25) of about 100,000 voxels and a multiples comparison correction, the significance value is 0.0000005 (0.05/100,000), which is a fairly conservative approach. Our compromise is a p-value of 0.000045 with a cluster size of 4 voxels. In addition, if the expected areas were not found the threshold value was lowered in 0.01 steps and the maximum correlation value of the area was detected. In this case, these areas were excluded from future analysis.

### 2.4. MEG recordings

The MEG experiments were performed previously to the MRI examinations. Cortical somatosensory and motor evoked fields (SEF and MEF) were continually recorded using a whole-head MEG system (MAGNES 3600 WH, 4-D Neuroimaging, San Diego, CA, USA) with 248-magnetometers which was confined in a magnetically shielded room (Vacuumschmelze, Hanau, Germany). MEG signal was acquired at a 678 Hz sampling rate using an online high-pass filter of 0.1 Hz and low-pass filter of 200 Hz. In addition to passive shielding, the online noise cancellation of the MEG system was applied by using reference channels (6 magnetometers and 5 gradiometers) to reduce ambient field.

During MEG data acquisition, the subject was in a supine position with the head placed stable by means of a neck rest. In order to achieve a good co-registration of MEG data and anatomical MRI data, we used 5 small coils placed on the surface of the patients head at left pre-auricular point, right pre-auricular point, nasion, Cz (Vertex) and inion. Digitization of reference points coils and head surface was done using the MEG-integrated SPI (sensor position indicator) with a 3D-tracking system (Polhemus, Colchester, VT, USA).

Motoric and somatosensory MEG recordings were performed in the same session following the comprehensive sensorimotor protocol (CSSMP) where external mechanic (sensoric) stimulation serves as a cue for subjects’ movements as described previously [28]. The experimental setup in short: Prior to the motor measurements the patients were instructed to repeatedly flex and extend the second, third, fourth and fifth finger (Exercise 1) or the foot and toes (Exercise 2) of a designated side. Healthy volunteers and patients were asked to refrain from any other motor actions. Following a short somatosensory trigger stimulus delivered by a pneumatic tap (17 lb/in^2^) to the tip of the subject’s index of the opposite hand via a balloon diaphragm (1 cm in diameter). Stimulus duration was 40 ms, interstimulus interval was 3.6 s, and stimuli and subsequent movement task were repeated 300 times to allow recording a sufficient number of trials to be averaged during MEG data evaluation. The somatosensory trigger stimulus was used instead of a visual trigger in order to avoid eye movement artefacts. The patients were instructed to move 4 fingers (digits 2 to 5) of the hand with a sudden onset of motion and as fast as possible to the heel of hand. Immediately afterwards, the patients had to open their hands again, in order to revert to the starting position. An electromyogram (EMG) was recorded via a pair of electrodes fixed to the lower arm muscles. This EMG signal was used to define the onset of the fast finger movement. The experiment was performed for both sides of the body.

All subjects were instructed to avoid any other motor actions like blinking, swallowing or moving other parts of the body as effectively as possible. The subjects and their finger movement were supervised using a camera installed in the shielded room. As the somatosensory stimulus was triggered via a stimulation PC, the evaluation of the somatosensory evoked fields of the forefinger of the other hand could be analyzed, too.

### 2.5. MEG data evaluation

All 300 trials of each MEG experiment were inspected visually by an experienced MEG investigator for magnetically artefacts (originating from the patient or external noise) and trials exhibiting artefacts were excluded from the study. All remaining trials (typically around 280) were averaged using the onset of the EMG-signal for motor function and the trigger signal from the stimulation PC for somatosensory function, respectively. The averaged data were filtered by band pass of 0.3 to 45 Hz.

Further MEG data analysis was done using the brainstorm software package (V3.4) [29]. Three different methods were used for MEG-based localization of SM function: (**i**) current density map with deep weighting using minimum norm estimate (MNE) [30]; (**ii**) dynamical Statistical Parametric Mapping (dSPM) [31], and (**iii**) standardized LOw Resolution brain Electromagnetic TomogrAphy (sLORETA) [32]. All three methods are based on minimal modelling to estimate distributed sources and are applicable on complex datasets or at high noise levels [33,34]. In MNE [35], maxima of inverse solutions were shifted towards the sensors [36,37]. Deep weighting and noise normalization were used for compensation. Deep weighting was done by normalizing all sources in the model using a measure of the overall amplitude. A minimization of the localization errors was achieved by normalization of rows of the lead field matrix [31]. Normalization of noise level was done by dividing the estimated current at each source location by an estimate of the noise at this location. In dSPM this was done by applying the inverse operator to the signal covariance matrix. In sLORETA the diagonals of the model resolution matrix were used [32]. In the case of a single dipole source, sLORETA was able to fit exactly to a single dipole field distribution [32]. The noise covariance data were calculated from baseline data in the pretrigger interval of 500–1000 ms. Individual brain MRI data (T1w MPRAGE) were used in all participants, to consider for lesion-induced changes in brain anatomy. BrainSuite (version 15c) was utilized to extract the individual brain structure data [38].

### 2.6. Multimodal localization of cortical sensorimotor function

The following cortical areas in the motor network were evaluated: (**i**) M1, located in the sulcus centralis (in Brodman area 4, BA 4); (**ii**) premotor area (PMA) in the sulcus precentralis (in BA 6); and (**iii**) supplementary motor area (SMA) in the fissura longitudinalis cerebri (in BA 6). The evaluated cortical areas in the cortical somatosensory network were: (**i**) the primary somatosensory cortex (SI) in the sulcus centralis (in BA 3), and (**ii**) the secondary somatosensory cortex (SII).

The fMRI-based cortical localization maps were adjusted with a fixed significance threshold (correlations > 0.3 with p < 0.000045) [24]. MEG-based cortical localization maps were individually threshold adjusted, i.e. a general threshold for all patients was not applicable. Both fMRI- and MEG-based cortical localizations were displayed on the anatomical 3D T1w MRI data set. The position of maxima and centroids of the fMRI- and MEG-based cortical localizations of SM function (M1, PMA, SMA, SI, and SII), respectively, were determined. The distances of the maxima and centroids, respectively, between the fMRI localizations and the MEG localizations obtained with the three different MEG data localization methods (MNE, dSPM, sLORETA) were calculated. Additionally, the shortest distances between the border of the fMRI-based cortical localizations and the tumor border on both 3D CE T1w MRI and 3D native T1w MRI were determined. This distance was not determined for the MEG results because the MEG-based cortical localization maps had to be adjusted with individual thresholds, which would yield to non-reliable results.

SEF and MEF latencies were defined as the time interval between the somatosensory stimulus trigger signal and the EMG signal, respectively, to the maximum brain activity of the evoked response shown in the corresponding brain area (SI, SII, M1, PMA and SMA). The MEG localization results were displayed on an anatomical 3D MRI data.

### 2.7. Statistical analysis

Mean values, standard deviations (SD), minima, and maxima were calculated for latencies and distances using the statistical software package R (version 3.4.2). For group analysis assuming independent samples and absence of Gaussian distribution Mann-Whitney U tests (reorganization yes/no) were used. *P* values less than 0.05 were considered to indicate significance.

## 3. Results

From the 13 patients included in this study (Table 1), one patient (ID 1) did not tolerate fMRI and another (ID 13) declined MEG examination. In two patients (IDs 10, 11) with dental implants the quality of the MEG data was insufficient because of magnetic artefacts. Overall fMRI data, MEG recordings and MEG and fMRI data of SM function prior to lesion resection were analyzed for twelve, ten and nine patients, respectively.

### 3.1. fMRI-based cortical localizations of sensorimotor function

**Fig 1:**
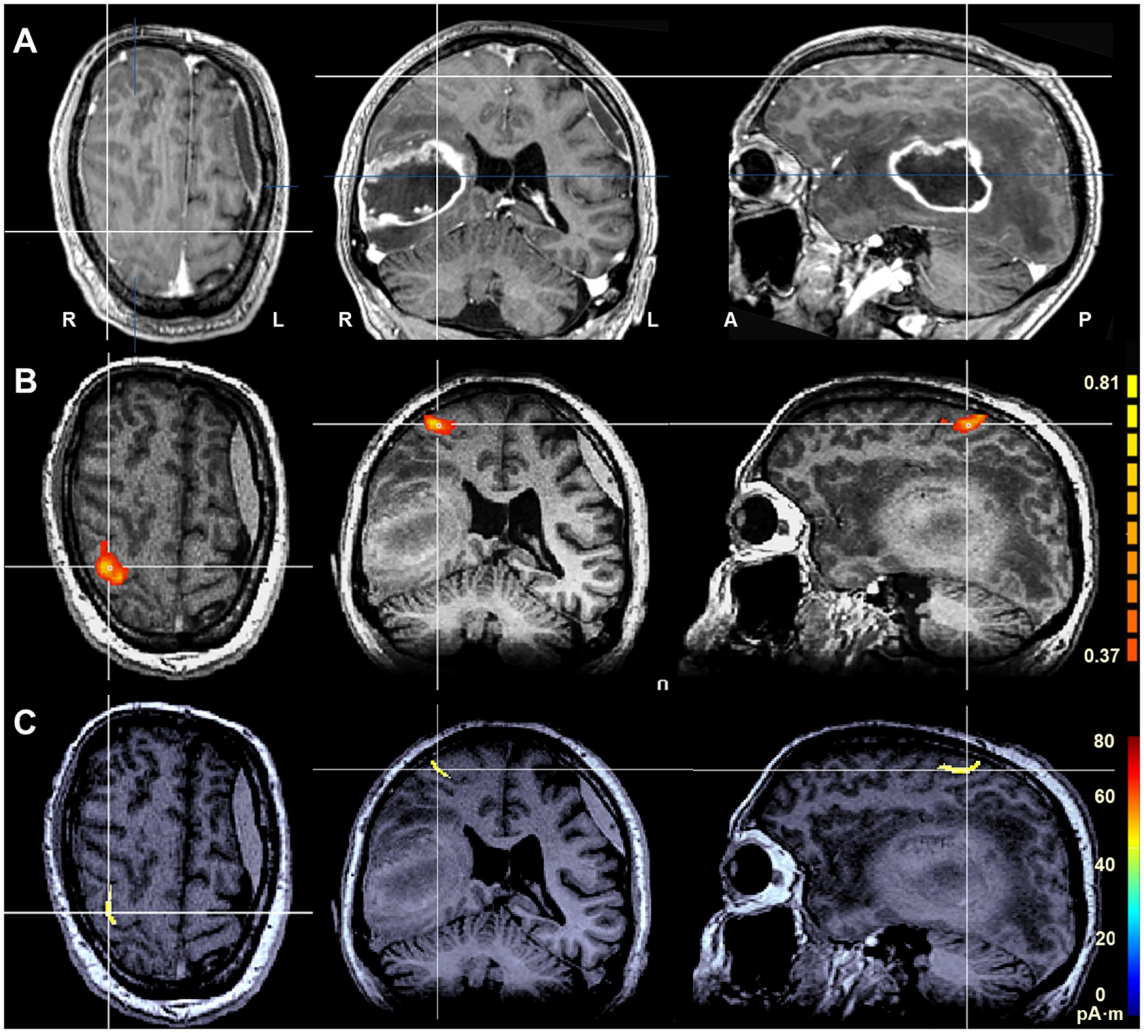
Normal M1 activation. Normal M1 activation in a glioblastoma patient (ID 5) without signs of cortical M1 reorganization; white lines indicating the slice intersections, color coding bars on right-hand side of panels B and C indicate correlation values and magnetic field strength, respectively. (A) Cranial contrast-enhanced T1w MRI in axial, coronal and sagittal orientation (left to right). (B) fMRI localization of M1 superimposed onto a native T1w MRI. (C) MEG localization of M1 superimposed onto a native T1w MRI. fMRI- and MEG-based cortical localizations show a high degree of spatial congruency.

**Fig 2:**
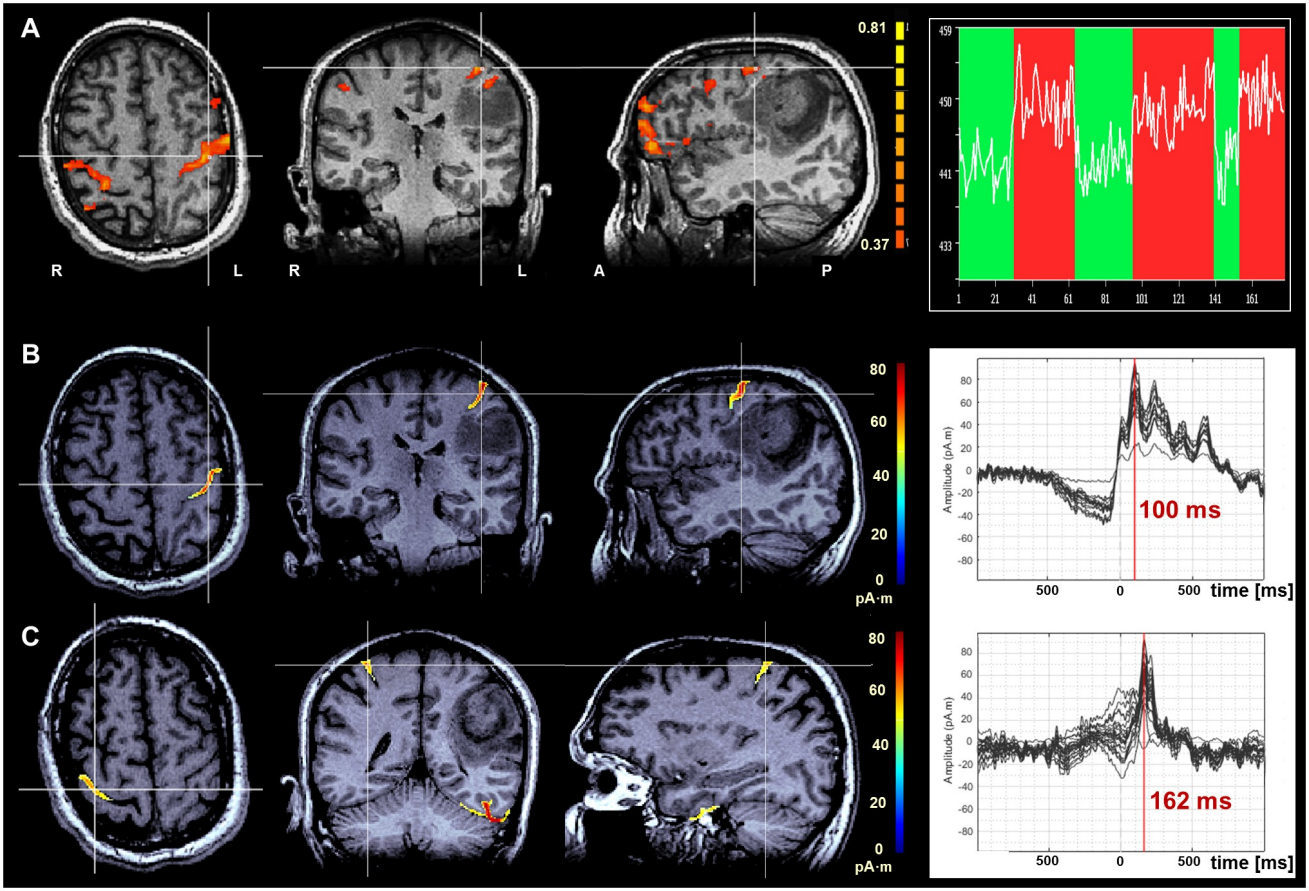
Cortical reorganization of the M1 activation. Cortical reorganization of the M1 activation in patient with anaplastic astrocytoma (ID 4) and signs of cortical M1 reorganization. Axial, coronal and sagittal planes are ordered from left to right with white lines indicating the slice intersections; color coding bars on right-hand side of the sagittal planes in A and B/C indicate correlation values and magnetic field strength, respectively. The diagrams on right-hand side of the composite show the block paradigm with the fMRI signal intensities in the selected voxel in A and the time courses of the magnetic field strength within the MEG data in the areas with M1 activation in B/C. (A) fMRI localization of M1 superimposed onto a native T1w MRI. (B/C) MEG localization of M1 superimposed onto a native T1w MRI. The latency difference between ipsi- (B) and contralateral (C) M1 activation was 62 ms. fMRI- and MEG-based cortical localizations show a high degree of spatial congruency in the between the contralateral, normal (B) and ipsilateral, reorganized (C) M1 areas.

In the fMRI mode tasks involving the contralesional hand revealed cortical activations in M1, SMA and SI / SII areas in all 12 patients. PMA activation, however, was apparent in only 11 of the 12 patients. More specifically fMRI findings were as follows:

1. M1: Seven patients showed normal M1 activation only ipsilateral to the lesion without indication for reorganization (Fig 1B). In contrast five patients (IDs 2, 4, 6, 12, 13) showed an additional M1 activation contralateral to the lesion indicative of M1 reorganization (Fig 2A) which will be described in greater detailed in 3.4.
2. SMA: In motor tasks (MT) involving the contralesional hand all 12 patients displayed SMA activity ipsilateral to the lesion without indication for reorganization. Notably the SMA area is located on the mesial surface of the hemisphere and thus the resolution of both fMRI and MEG may not suffice to reliably detect a shift to the contralateral homotopic area.
3. PMA: Eleven of 12 patients showed PMA activity ipsilateral to the lesion. Only ID 5 showed no BOLD response in the ipsi- or contralateral PMA at the default threshold of 0.3 and a cluster size of 4 voxels (p < 0.000045). However, using the same cluster size this area becomes apparent at a threshold level of 0.21 (p < 0.0047). Conversely keeping the threshold at 0.3 whilst lowering the cluster size to 2 voxels has the same result; using a single PMA voxel the maximum correlation is 0.34 (p = 0.000003). This constellation could be due to a large tumor with pronounced edema leading to compression of adjacent brain areas and degradation of the fMRI signal. Nevertheless this ID 5’s PMA was excluded from further analysis.
4. SI / SII: In all 12 patients sensory stimulation of the contralesional hand highlighted the primary and secondary sensory area ipsilateral to the lesions, indicative of no reorganization in the cortical somatosensory network.

### 3.2. MEG-based cortical localizations of sensorimotor function

In the MEG mode tasks involving the contralesional hand revealed cortical activation patterns for M1, SMA, PMA and SI / SII essentially identical to those found in fMRI mode (Fig 1C).

1. M1: All 10 patients showed the expected activation of the primary motor area. MEG data were available for only four (IDs 2, 4, 6, 12) of the five patients with fMRI-based signs of reorganization. MEG data in these four patients showed additional M1 activations contralateral to their lesions (Fig 2B and 2C) consistent with M1 reorganization. Details of the M1 reorganization and specific latency times are given below.
2. SMA: All 10 patients revealed SMA activity only ipsilaterally to the lesion for MT involving the contralesional hand. In accordance to the fMRI results there are no signs of SMA reorganization.
3. PMA: All 10 patients including ID5 without BOLD response revealed PMA activity ipsilateral to the lesion for MT involving the contralesional hand.
4. SI / SII: In all 10 patients sensory stimulation of the contralesional hand revealed the expected activations of cortical somatosensory areas. In accordance to the fMRI results there are no signs of SI / SII reorganization.

In these 10 patients, latencies of the SEF responses post stimulation of the index fingertip ranged from 54 to 66 ms and 87 to 111 ms in SI and SII, respectively.

The three postprocessing methods (MNE, dSPM, sLORETA) yielded different cortical localizations of the maxima and the centroids of the above areas in the millimeter range, with smaller differences observed in the sLORETA / dSPM pairs compared to the sLORETA / MNE and MNE /dSPM pairs (Table 2).

**Table 2:** Spatial comparison of three different MEG localization methods.

	Distance between two MEG localization methods [mm]					
Subjects	MNE/dSPM (Maxima)	MNE/sLORETA (Maxima)	sLORETA/dSPM (Maxima)	MNE/dSPM (Centroid)	MNE/sLORETA (Centroid)	sLORETA/dSPM (Centroid)
	**Motor Activation**					
Patients	12 ± 6 (0–25)	9 ± 6 (0–28)	7 ± 5 (0–19)	8 ± 5 (0–20)	6 ± 5 (0–20)	4 ± 3 (0–10)
Volunteers	13 ± 4 (1–17)	11 ± 5 (2–19)	9 ± 6 (0–18)	9 ± 4 (3–17)	8 ± 4 (1–16)	6 ± 5 (1–18)
	**Somatosensory Stimulation**					
Patients	11 ± 4 (1–18)	7 ± 4 (0–16)	6 ± 5 (0–17)	6 ± 4 (1–17)	4 ± 3 (0–10)	4 ± 4 (0–19)
Volunteers	13 ± 4 (3–19)	7 ± 5 (0–16)	6 ± 5 (0–15)	6 ± 3 (2–13)	4 ± 2 (1–9)	3 ± 2 (0–8)

All values mean ± SD (min—max) are Euclidian distances of MEG localizations between two of three methods using maxima and centroids, respectively, for motor localizations averaged over M1, PMA, and SMA, as well as for somatosensory localizations averaged over SI and SII.

### 3.3. Spatial comparison of fMRI and MEG localizations

The localizations of cortical SM areas showed good correspondence between fMRI and MEG.

For the motor localizations (n = 26) the mean distances ± SD between the centroids of the two modalities were 8 ± 4 mm, 8 ± 4 mm and 9 ± 4 mm (Table 3) with MEG-based centroids mapping within the fMRI-defined boundaries in 68%, 55% and 45% using MNE, sLORETA and dSPM, respectively. Patients’ MEG centroids outside the fMRI-defined boundaries localized at distances of 6 ± 3 mm, 7 ± 3 mm and 8 ± 2 mm using dSPM, sLORETA and MNE, respectively (Table 4).

**Table 3:** Spatial comparison of fMRI and the three different MEG localization methods.

	Distance between fMRI and MEG [mm]					
Subjects	MNE (Maxima)	dSPM (Maxima)	sLORETA (Maxima)	MNE (Centroid)	dSPM (Centroid)	sLORETA (Centroid)
	**Motor Activation**					
Patients	9 ± 5 (2–18)	11 ± 4 (3–20)	11 ± 4 (5–18)	8 ± 4 (2–15)	9 ± 4 (2–15)	8 ± 4 (1–15)
Volunteers	12 ± 6 (3–19)	14 ± 5 (6–20)	14 ± 5 (8–21)	9 ± 5 (1–14)	12 ± 6 (2–22)	12 ± 5 (6–21)
	**Somatosensory Stimulation**					
Patients	15 ± 5 (10–20)	13 ± 10 (5–24)	9 ± 5 (7–15)	11 ± 4 (6–15)	11 ± 6 (7–18)	8 ± 2 (7–11)
Volunteers	18 ± 4 (15–23)	12 ± 6 (7–19)	14 ± 7 (7–22)	12 ± 2 (11–15)	8 ± 3 (5–10)	8 ± 3 (5–10)

All values mean ± SD (min—max) are Euclidian distances between fMRI and MEG using maxima and centroids, respectively, for motor localizations averaged over M1, PMA, and SMA, as well as for somatosensory localizations averaged over SI and SII.

**Table 4:** Spatial comparison of the fMRI off-border localization and the three different MEG localization methods.

	Distance between fMRI off-border and MEG [mm] (Only cases with MEG outside of fMRI border)					
Subjects	MNE (Maxima)	dSPM (Maxima)	sLORETA (Maxima)	MNE (Centroid)	dSPM (Centroid)	sLORETA (Centroid)
	**Motor Activation**					
**Patients**^†^: mean Volume 1.4 cm³	32% 8 ± 2 (0–11)	50% 7 ± 3 (0–14)	55% 7 ± 3 (0–15)	32% 8 ± 2 (0–10)	54% 6 ± 3 (0–12)	45% 7 ± 3 (0–12)
**Volunteers**^†^: mean Volume 2.8 cm³	56% 7 ± 3 (0–12)	44% 7 ± 3 (0–11)	44% 8 ± 3 (0–11)	33% 4 ± 1 (0–5)	33% 8 ± 3 (0–11)	44% 7 ± 3 (0–9)
	**Somatosensory Stimulation**					
**Patients**^†^: mean Volume 1.7 cm³	50% 12 ± 5 (5–15)	33% 5 ± 1 (4–5)	33% 4 ± 3 (1–8)	33% 12 ± 3 (9–14)	33% 2 ± 1 (1–3)	50% 2 ± 2 (1–4)
**Volunteers**^†^: mean Volume 1.5 cm³	33% 9 ± 2 (8–11)	17% 5 ± 0 (5–5)	17% 8 ± 0 (8–8)	33% 8 ± 4 (4–11)	33% 4 ± 2 (2–6)	33% 4 ± 1 (3–4)

All values mean ± SD (min—max) are Euclidian distances between fMRI border and MEG using maxima and centroids, respectively, for motor localizations averaged over M1, PMA, and SMA, as well as for somatosensory localizations averaged over SI and SII.

^†^percentage of patients/volunteers with MEG outside of fMRI activation volume.

For the somatosensory localizations (n = 18) the mean distances ± SD between the centroids of the two modalities were 8 ± 2 mm, 11 ± 4 mm and 11 ± 6 mm for sLORETA, MNE and dSPM, respectively (Table 3); the MEG-based centroids mapped within the fMRI-defined boundaries in 67%, 67% and 50% using dSPM, MNE and sLORETA, respectively. Patients’ MEG centroids outside the fMRI-defined boundaries localized at distances of 2 ± 1 mm, 2 ± 2 mm and 12 ± 3 mm using dSPM, sLORETA and MNE, respectively (Table 4).

### 3.4. Motor cortex reorganization

M1 reorganization was suggested on the basis of fMRI and MEG data in 5 of 12 (IDs 2, 4, 6, 12, 13) and in 4 of 10 patients (IDs 2, 4, 6, 12), respectively (Fig 2).

In the 5 patients (all gliomas) with M1 reorganization the distances between the fMRI-based M1 boundaries and the native T1w-segmented tumor border ranged from 0 to 4 mm which differs significantly from those in patients without M1 reorganization ranging between 5 to 26 mm (*p* = 0.025; Table 1).

**Fig 3:**
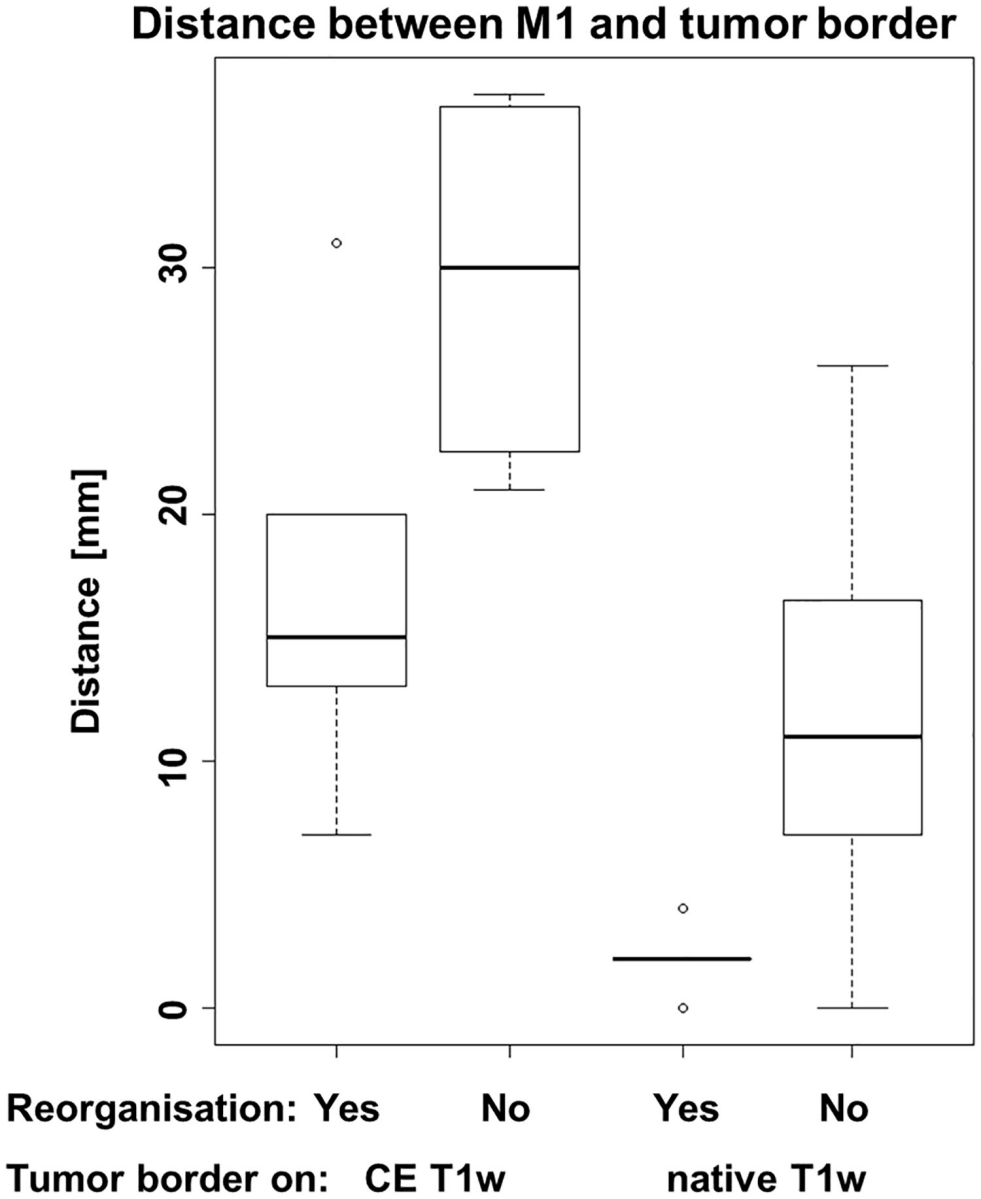
Distances between M1 and the tumor border. Distances between the border of the cortical M1 activation in fMRI and tumor border on 3D T1w MRI (contrast enhanced, CE and native) for patients with and without reorganization of the M1 area.

Interestingly, the two patients with well-defined AVM/H (ID 7 and 8) showed no M1 reorganization albeit at minimal M1-to-tumor distances (2 and 0 mm, respectively). The distance between the borders of the fMRI M1 localization and the lesion border on the CE T1w MRI ranged between 7 to 31 mm in the patients with M1 reorganization, and between 24 and 36 mm in the patients without indications for cortical M1 reorganization. This difference, however, was not statistically significant (*P* = 0.063; Fig 3).

**Fig 4:**
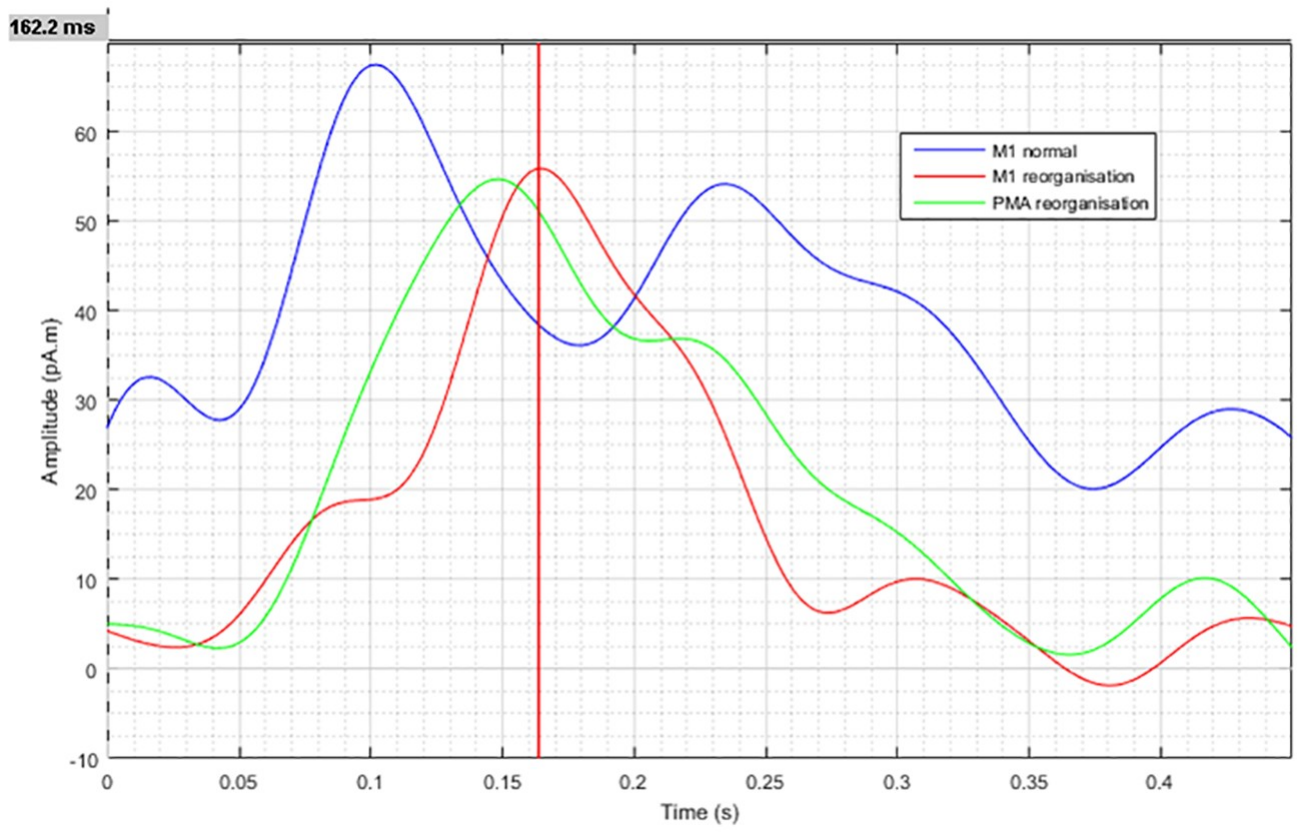
M1 time lines of cortical M1 reorganization. M1 time lines of the magnetic field strength (calculated by MNE) in a patient with anaplastic astrocytoma (ID 4) and signs of cortical M1 reorganization. The blue and red traces depict the magnetic field strength originating from the contralateral, normal and ipsilateral, reorganized M1 areas with a delay of 62ms. The green traces depict the magnetic field strength originating from the contralateral ipsilateral PMA area.

Investigation of the latency times of the MEG-based M1 activation on ipsilateral and contralateral (reorganized) side to the lesion revealed that the ipsilateral M1 activation was on average 60 ± 8 ms (52–67 ms) earlier than the contralateral M1 activation (Fig 4).

### 3.5 Clinical correlations and follow-up

Seven (IDs 3, 5, 6, 9, 10, 11, 12) of 13 patients had preoperative SM deficits which in five of them resolved postoperatively. The tumors of two patients with persistent SM deficits were at 5 mm distances to M1 (ID 9) and PMA (ID 10), respectively, yet infiltrated the PT and BA6, respectively.

Five (IDs 1, 2, 4, 7, 13) of 13 patients were free of pre- and post-operative SM deficits; three (ID 2, 4, 13) of them had M1 reorganization although suffering from infiltration or compression of the PT or MC and the other two showed no infiltration or compression of the PT or MC.

One patient with AVM/H (ID 8) had a hemiparesis on the right side, one month later the patients SM deficits decrease to a discrete hemiparesis. The AVM/H was located in the postcentral gyrus and at the surgery the areas for proprioception and sensory function were damaged.

Pre-operative PT or MC compression was present in seven patients (IDs 2, 3, 4, 5, 6, 11, 12), two of them (IDs 2, ID 4) showing M1 reorganization had no SM deficits.

Tumor infiltration was present in six patients (IDs 3, 6, 9, 10, 12, 13), three of them (IDs 6, 12, 13) showing M1 reorganization had no post-operative SM deficits. In contrast, two of the patients without M1 reorganization (IDs 9, 10) had postoperative SM deficits and one patient without M1 reorganization had postoperative no SM deficits.

Four (IDs 2, 6, 12, 13) of five patients with showing M1 reorganization had resections in MC. ID 2 nearby tongue area approx. 2 cm to the hand area, ID 6 nearby leg area approx. 2 cm to the hand area, ID 12 nearby tongue area approx. 2 cm to the hand area and ID 13 about 1 cm to the hand area.

## 4. Discussion

Functional MRI is used regularly to depict brain function near gliomas for preoperative surgical planning. But it’s a matter of discussion, whether BOLD areas actually represent neuronal activity, whether disappearance of a BOLD signal is equal to non-existent neuronal activity and whether reorganization of a M1 area actually exists. To investigate these unresolved scientific questions, we used a combination of fMRI and MEG for the characterization of cortical SM functions in the vicinity of cerebral gliomas and brain AVM/Hs.

We found high concordance of both imaging modalities concerning the spatial resolution and our results indicate that reorganization of motor areas in the vicinity of such brain lesions plays an important role.

In our study, MEG and fMRI provided quite similar localization results and confirm of each other: Overall 60% of the MEG localizations (maxima and centroids) mapped within the fMRI activation volumes. The remaining MEG maxima and centroids were localized at mean distances of 3.8 mm for SEF with sLORETA and 7.4 mm for MEF with MNE outside from the fMRI volume boundaries (more detail in Table 4).

In SM tasks a high degree of colocalization was found for fMRI and MEG activation in three healthy volunteers and thirteen patients. The spatial variability between fMRI and MEG activation sites (maxima and centroids) for the MT and sensory tasks (ST) of approximately 10 mm (more detail in Table 3) in our study corresponds with those of a previous study [39] which reported 10 ± 5 and 15 ± 5 mm for the motor and ST, respectively. The smaller variability of the somatosensory localizations in our data may well be due to our use of more effective algorithms or the other group of subjects.

Generally, the following reasons or their combinations could be responsible for the small localization differences observed: (**1**) fMRI and MEG reflect the different neurophysiological phenomena, namely changes in the regional tissue oxygenation and the electrical activity in dendrites of the neurons; (**2**) Limited ability to record currents perpendicular to the skull in axial coil MEG system; (**3**) Different deep weighting procedures, noise normalization methods and the numeric solution of the inverse problem with localization algorithms of MEG data are used; (**4**) Complexity of field distribution compounded by a multiplicity of simultaneously active sources increase the MEG localization error; (**5**) Movement- and magnetic- artefacts; (**6**) Errors in fusing the functional data with the T1w MRI anatomical data set.

More specifically the different MEG localization algorithms provide different results which can be explained by the effects of deep weighting and noise normalization used in dSPM and sLORETA. For example the mean deviation between the MNE and dSPM maxima of 12 mm differs considerably from the one between the sLORETA and dSPM centroids of 4 mm. As expected from theoretical considerations dSPM [31] and sLORETA [32] provide the most closely corresponding localizations, followed by the MNE/sLORETA and MNE/dSPM pairs. This holds for both motor and sensory localizations in patients as well as in volunteers. Theoretically sLORETA should provide better fits for the mostly dipolar SEF sources, whereas MNE should be better suited for the more complex MEF fields. This expectation is borne out by the comparison of the maxima and centroids of fMRI and MEG as we observed the best intermodal spatial correspondence for MT and ST using MNE and sLORETA, respectively. For MT, MNE localizes the most MEG data points within the corresponding fMRI volumes, whereas for ST, dSPM provides the best spatial correspondence. The latter difference could well be due to the limited number of tests in this study.

Our cohort with brain tumors patients supports strongly the concept of M1 reorganization. A previous combined fMRI/MEG study of 325 consecutive patients suffering from a range of diseases including non-lesional epilepsy, stroke, and a variety of brain lesions found signs of motor cortex reorganization in about 14% yet without statistical significance of a specific disease association [7]. Our study allows consideration of motor cortex reorganization in patients with high grade gliomas which were localized at a distance of 4 mm or less from the M1 area. This is indicative of function-conserving activities once cortical motor functions are affected by tumor cell infiltration. This is in accordance with earlier fMRI investigations showing ipsilateral activation in 31 of 87 patients with centrally located tumor and a statistically significant increase of motor cortex reorganization in patients with glioblastoma WHO grade IV [11]. In summary, both the presence of a malignant tumor and the proximity to the motor cortex are likely inducers of functional reorganization, reflected in contralesional cortical activation.

In another fMRI study of cortical reorganization together with intraoperative mapping or patient surgery outcome a false positive rate for fMRI-detected reorganization was reported in seven of 23 patients (30%) [12]. However, it is important to consider some important aspects regarding this study: (**1**) three of them were language areas; (**2**) three of the other four patients had tongue problems. It is well known, however, that motor tongue areas are typically bilateral and a resection of one of these areas in nearly all patients only causes temporary problems in tongue movement [40]; (**3**) Only one case out of these four refers to supplementary motor area (none M1 area) and might be comparable to our study: this is in a patient with an AVM/H, possibly associated with a hemorrhage. As a consequence of this disturbance of the BOLD-Effect may be possible. Furthermore, the fMRI motor reorganization results in our study were confirmed by MEG which detects neuronal activity directly, whereas fMRI detects changes of the blood flow oxygenation.

Five of the 13 patients showed cortical M1 reorganization in fMRI, evident also in the four patients for whom MEGs were recorded. None of the patients with M1 reorganization had postoperative deficits although tumor resections were close to (in 4) or immediately adjacent to the motor area (in 1). In summary, all patients with M1 reorganization had a favorable postoperative outcome, even though some of their tumors had already infiltrated the PT or MC. This argues for a positive effect of M1 reorganization on motor function although functional contributions of the remaining contralateral motor area cannot be excluded.

To better understand the physiology of M1 reorganization it may be relevant that on average reorganized (ipsilateral) MEG activation was found delayed by around 60 ms to the normal contralateral activation. This is consistent with prior observations [23] in which isohemispheric signal delays between M1 to PMA of around 40 ms were reported in healthy subjects. Reorganization can be reasonably expected to result in slower processing compared to healthy brain activity, as motor area reorganization may result from gradually utilizing the functionality of existing cortex areas for movement integration of limbs of both body sides. Brain tumors which only slowly progress may well grant the sufficient time for such neuronal development to proceed effectively.

In contrast, no somatosensory reorganization (ipsilateral to the stimulation) was observed in our data, neither in fMRI nor MEG. This could be expected for two reasons: Firstly, the patients in our cohort showed mostly tumoral motor cortex proximity and somatosensory cortex might have been less affected. Secondly, earlier reports have shown that somatosensory activation remained restricted to the lesioned hemisphere [7,41].

No pre- and postoperative SM deficits were observed in five patients, three of them with M1 reorganization had, no SM deficits despite infiltration and/or compression. The other two of them showed neither infiltration nor compression. In the six patients tumor infiltration in MC were observed, are three of them showed M1 reorganization and had no SM deficits whereas two without reorganization had pre- and postoperative SM deficits. Summarized, all patients with reorganization had positive good outcome with regard to SM deficits. The connection between M1 reorganization and better outcome regarding SM deficits seems to be obvious.

Pre-operative PT or MC compression was observed in seven patients, after the operation and the associated decompression a significant improvement of the SM function occurred. We found with default settings for fMRI postprocessing no fMRI activation in the PMA in patient ID 5 with a very large tumor and pronounced edema. This could lead to compression of the brain areas and associated changes in the fMRI signal. For that reason, it’s important to adapt threshold and cluster size settings for compensation of lesion induced BOLD signal attenuations.

Attention should be focused on the general discussion having started about the possible replacement of invasive presurgical procedures by non-invasive ones, like fMRI and MEG launched by Papanicolaou et al. [42]. They conclude that there are no longer compelling reasons for opting for invasive mapping in many if not the most cases provided that the non-invasive methods are available.

## 5. Conclusion

The comparison of localization results of fMRI and MEG reveals a high degree of spatial congruence in healthy volunteers and preoperative patients with cerebral lesions. This multimodal approach of functional mapping demonstrates the occurrence of M1 reorganization in close proximity to high-grade gliomas, excluding neurovascular uncoupling by MEG confirmation. The superior temporal resolution of MEG allowed detecting an activational delay between the contralateral and the ipsilateral M1 areas which may contribute to a better understanding of the reorganization process.

The clinical use of non-invasive functional mapping yields a reduction of post-operative motor deficits. Future works will be necessary to use its full potential to enable more radical resections leading to an improved patient outcome and postoperative quality of life.
